# Data Fusion Based on Subspace Decomposition for Distributed State Estimation in Multi-Hop Networks

**DOI:** 10.3390/s19010009

**Published:** 2018-12-20

**Authors:** Álvaro Rodríguez del Nozal, Pablo Millán, Luis Orihuela

**Affiliations:** Departamento de Ingeniería, Universidad Loyola Andalucía, 41014 Sevilla, Spain; pmillan@uloyola.es (P.M.); dorihuela@uloyola.es (L.O.)

**Keywords:** distributed estimation, LTI-systems, kalman-filtering, data fusion, multi-hop networks

## Abstract

This paper deals with the problem of estimating the distributed states of a plant using a set of interconnected agents. Each of these agents must perform a real-time monitoring of the plant state, counting on the measurements of local plant outputs and on the exchange of information with the rest of the network. These inter-agent communications take place within a multi-hop network. Therefore, the transmitted information suffers a delay that depends on the position of the sender and receiver in a communication graph. Without loss of generality, it is considered that the transmission rate and the plant sampling rate are both identical. The paper presents a novel data-fusion-based observer structure based on subspace decomposition, and addresses two main subproblems: the observer design to stabilize the estimation error, and an optimal observer design to minimize the estimation uncertainties when plant disturbances and measurements noises come into play. The performance of the proposed design is tested in simulation.

## 1. Introduction

In the last decade, the development of intelligent sensors and actuators, endowed with communication and computational capabilities, has fostered their integration in a variety of large-scale plants, especially in cyber-physical systems and cyber-physical systems of systems [1]. These intelligent devices are entangled with physical systems and interact in real-time with them, receiving measurements, making calculations, and taking decisions in a continuous manner. To perform these tasks, the sensors need to exchange measurements of the system outputs, which are very often geographically distributed and are only a small subset or linear combination of the whole system state. This exchange of information makes it possible to reconstruct the complete state distributedly using the so-called distributed estimation algorithms, which have recently attracted much attention from the control community.

Although distributed estimation architectures offer several advantages, such as scalability, flexibility, fault tolerance, and robustness [2], it is also true that the complexity associated with these problems makes them harder to solve. This is due to the fact that none of the nodes manage all the state measurements of the plant and thus they require an exchange of some state information, sometimes experiencing delays and communication dropouts that complicate the maintenance of the observer’s stability. Consequently, a remarkable research effort has been made in this field and important contributions to the topic can be found in the literature adapted to different considerations, for example the system modeling or the communication setup. Attending to the former feature, several approaches have been published for the case of noiseless/unperturbed models, where typically the main objective is the asymptotic stabilization of the estimation errors. For instance, in [3,4], reduced-order observers are designed based on coordinate transformations. However, although both observers can run distributedly, their designs need to be carried out in a centralized way. Following the same line, in [5], different matrix transformations are introduced to divide the observable and unobservable subspaces of each agent, resulting on an observer design that can be carried out distributedly. However, the design method does not allow tuning of the convergence rate and the distributed design requires a huge amount of information to be exchanged. Furthermore, in [6], a distributed observer whose design can be tackled in a distributed way is presented. Nevertheless, its performance relies on some parameters whose tuning remains unclear.

When considering perturbed or noisy scenarios, the observer design must guarantee, to some extent, the stability of the estimation error under the perturbed scenario and, typically, optimize some metric of the estimation uncertainties. Maybe one of the most well known approaches for this kind of problem is the distributed Kalman filter (DKF), first presented in [7,8]. Within this approach, Gaussian disturbances and noises are both considered, and the observer is designed with the aim of minimizing the error covariance of the estimates taking into account measured outputs and information received from neighboring nodes. The interested reader can find more details of this approach in [9], an experimental performance evaluation. Another approach to the same problem can be found in [10], where the authors relay a state decomposition in order to minimize the information exchange during the estimation phase. However, the huge amount of required information complicates the distributed design. Other approaches to this problem are based on distributed consensus techniques, for which the interested reader is referred to [11], or distributed guaranteed estimation, in which noises and disturbances are assumed to belong to bounded sets and the concept of error covariation replaces that of error covariance in the Gaussian approach [12].

Finally, a productive research field with straightforward applications to the estimation problem is data fusion. Data fusion is the process of integrating multiple data sources to produce more consistent, accurate, and useful information than that provided by any singular data source [13]. Data fusion strategies have been implemented widely in a broad variety of applications, such as body sensor networks [14], optical and radar remote sensing [15], and greenhouse monitoring [16]. Most of the approaches found in the literature implement data fusion strategies in which the information flows through the network fast enough to be available everywhere in one sample time. More precisely, the information taken by all the agents is collected at every sample step. In that way, the main problem tackled is to present conditions under which the observer reaches consensus among the agents, not diverging in their decisions (see for instance [17] or [18]).

This paper deals with the problem of estimating the state of a perturbed plant by a network of agents executing a distributed data-fusion-based algorithm. Each agent knows the model of the plant and can measure some system outputs affected by noise. The rest of the necessary outputs are obtained through the exchange of information with neighboring agents. Different from the approaches mentioned before, this paper considers that the agents communicate through a multi-hop network, where data transmitted may take several sampling instants to reach its final destination. In other words, the communications are affected by graph-induced delays. Conversely to other approaches such as [19], we are not considering signal transmission delays or signal processing delays in the agents. The proposed observer structure decouples the state-space into several subspaces according to the observable modes considering the information received at each sample time. This exploits the idea that was previously presented by the authors in [20], where the number of agents was limited to two, and in [21], where a generalization to an arbitrary number of agents was adopted. However, neither of those papers considered the data-fusion scheme nor the disturbances and noises. The observer gains are designed in order to guarantee the stability of the distributed observer in spite of the presence of delays. Finally, a method to design the observer gains that minimizes the expected estimation error is presented. The main contributions of the paper are listed next:

- The introduction of a novel data-fusion-based observer structure able to be designed in a distributed fashion.
- Unlike the conventional data fusion approaches [17] or [18], the information is not required to spread through the network in a single sample time, thereby relaxing the requirements of the network.
- The observer design reduces the exchange of information with respect to other data fusion algorithms, due to the fact that the agents are not required to collect the information of every agent to reconstruct the whole state.
- In case of duplicated information, the proposed subspace decomposition allows the observer to be selective when deciding the agents who will be the source of the required data.

The paper is organized as follows. In Section 2, the problem is formally stated, together with some definitions and assumptions. Section 3 presents the observer structure. In Section 4 some properties useful for the consequent developments are introduced. Section 5 and Section 6 present methods to design the observer in order to guarantee the stability in the estimations and to minimize the expected estimation error when the system and the measurements made by the agents are affected by noise, respectively. In Section 7 some simulations examples are exposed. Finally, conclusions are drawn in Section 8.

**Notation** **1.** *A graph is a pair G=(V,E) comprising a set V={1,2,⋯,p} of vertices or agents, and a set E⊂V×V of edges or links. A directed graph is a graph in which edges have directions, so that if (j,i)∈E, then agent i obtains information from agent j. A directed path from node $i_{1}$ to node $i_{k}$ is a sequence of edges such as $\left(i_{1},i_{2}\right)$, $\left(i_{2},i_{3}\right)$, ⋯, $\left(i_{k-1},i_{k}\right)$ in a directed graph. The neighborhood of i, $N_{i}:=\left\{j:\left(j,i\right)\in E\right\}$, is defined as the set of nodes with edges incoming to node i. Given $\rho \in Z_{+}$, the ρ-hop reachable set of i, $N_{i,\rho }$, is defined as the set of nodes with a direct path to i involving ρ edges. Loosely speaking, the ρ-hop reachable set of agent i is comprised of those agents whose transmitted information takes ρ hops, or sampling instants, to reach to agent i. Note that the *1*-hop reachable set of i corresponds to the neighborhood of i and the *0*-hop reachable set of i matches with i. The operator col(·,·) stacks subsequent matrices into a column vector, e.g., for $A\in R^{m_{1}\times n}$ and $B\in R^{m_{2}\times n}$, $\mathrm{col}\left(A,B\right)={\left[\begin{array}{cc} A^{⊤} & B^{⊤} \end{array}\right]}^{⊤}\in R^{(m_{1}+m_{2})\times n}$. Let $I_{n}$ be the identity matrix of dimension n. Let E{·} denotes the expected value.*

## 2. Problem Formulation

Consider a set of agents V={1,2,⋯,p} connected according to a given graph G=(V,E), and intended to distributedly estimate the state of the following discrete-time linear time-invariant system:

(1) $$\begin{array}{c} x(k+1)=Ax(k)+w(k), \end{array}$$

(2) $$\begin{array}{c} y_{i}\left(k\right)=C_{i}x\left(k\right)+n_{i}\left(k\right), \end{array}$$

where $x\in R^{n}$ is the state vector, $A\in R^{n\times n}$ is the system matrix, $y_{i}\in R^{m_{i}}$ is the output locally measured by each agent *i*, $C_{i}\in \;R^{m_{i}\times n}$ is the corresponding output matrix and $w\in R^{n}$ and $n_{i}\in R^{m_{i}}$ are mutually independent Gaussian state and measurements noises, respectively, with covariance matrices $M\in R^{n\times n}$ and $R_{i}\in R^{m_{i}\times m_{i}}$.

We assume that the information flows slowly through the network, consuming one sample time to get from any agent to its neighbors. This is the case, for instance, of networks set up based on Zigbee [22].

The observation structure proposed in the next section relies on system transformations to the observability staircase form (see for instance Theorem 16.2 in [23]). Prior to introducing this structure, the following definitions are needed.

**Definition** **1.** *The ρ-hop output matrix of agent i, $C_{i,\rho }$, is a matrix that stacks the (ρ−1)-hop output matrix of agent i and the (ρ−1)-hop output matrices of its neighborhood, $N_{i}$. That is:*

$$C_{i,\rho }:=\left[\begin{array}{c} C_{i,\rho -1}\\ \mathrm{col}{\left(C_{j,\rho -1}\right)}_{j\in N_{i}} \end{array}\right],\;\forall \rho \geq 1,$$

*where $C_{i,0}:=C_{i}$.*

Intuitively speaking, the ρ-hop output matrix of agent *i*, $C_{i,\rho }$, is composed by its output matrix $C_{i}$ and the output matrices of all the agents *j* with a direct path to *i* involving ρ or less edges.

**Definition** **2.** *System *(1)* is locally detectable from agent i if pair $\left(C_{i},A\right)$ is detectable. System *(1)* is collectively detectable if every agent is able to detect the whole system with the information provided by the network. This is, for each agent i∈V, there exists a finite number of hops $\ell_{i}\in Z_{+}$ such that pair $\left(C_{i,\ell_{i}},A\right)$ is detectable.*

**Assumption** **1.** *We assume that system* (1) *is collectively detectable.*

There always exists a coordinate transformation matrix $\left[{\bar{V}}_{i,\rho }\;\;V_{i,\rho }\right]\in R^{n\times n}$ according to pair $\left(C_{i,\rho },A\right)$ such that the change of variable $\xi_{i,\rho }:={\left[{\bar{V}}_{i,\rho }\;\;V_{i,\rho }\right]}^{⊤}x\in R^{n}$ transforms the original state-space representation into the observability staircase form. Note that ${\bar{V}}_{i,\rho }\in R^{n\times n_{i,\rho }^{\bar{o}}}$ is composed by $n_{i,\rho }^{\bar{o}}$ column vectors in $R^{n}$ that form an orthogonal basis of the unobservable subspace of pair $\left(C_{i,\rho },A\right)$. Correspondingly, $V_{i,\rho }\in R^{n\times n_{i,\rho }^{o}}$ is an orthogonal basis of its orthogonal complement.

**Definition** **3.** *The ρ-hop unobservable subspace from agent i, denoted ${\bar{O}}_{i,\rho }$, is composed of all system modes that cannot be observed from the output locally measured by agent i and those measured by all the agents belonging to the s-hop reachable set of i, ∀s∈{0,⋯,ρ}. Equivalently, the ρ-hop unobservable subspace from agent i is the unobservable subspace related to pair $\left(C_{i,\rho },A\right)$ using the above coordinate transformation: ${\bar{O}}_{i,\rho }:=Im\left({\bar{V}}_{i,\rho }\right).$ The orthogonal complement of ${\bar{O}}_{i,\rho }$, with some abuse of notation, is denoted ρ-hop observable subspace from agent i, $O_{i,\rho }:=Im\left(V_{i,\rho }\right)$. We denote $n_{i,\rho }^{o}=dim\left(O_{i,\rho }\right)$.*

According to Definition 3, it is clear that:

(3) $$O_{i,\rho -1}\subseteq O_{i,\rho },\;\forall i\in V,\;\rho \geq 0.$$

where we consider $O_{i,-1}=\emptyset$. Then, the vectors of the “innovation” basis that generates $O_{i,\rho }\cap {\left(O_{i,\rho -1}\right)}^{⊥}$ can be stacked into a matrix $W_{i,\rho }\in R^{n\times n_{i,\rho }}$, where $n_{i,\rho }=n_{i,\rho }^{o}-n_{i,\rho -1}^{o}$, in such a way that:

(4) $$Im\left(W_{i,\rho }\right):=O_{i,\rho }\cap {\left(O_{i,\rho -1}\right)}^{⊥},\;\rho \geq 0,$$

Let us, to be selected later, define $\ell_{i}\in Z_{>0}$ as an arbitrary number of hops. From these definitions it is clear that for all $\rho \in \{0,\cdots ,\ell_{i}\}$ and all i∈V, it holds that

(5) $$\begin{array}{c} Im\left(V_{i,\rho }\right)=Im\left(\left[\begin{array}{cc} W_{i,\rho } & V_{i,\rho -1} \end{array}\right]\right), \end{array}$$

(6) $$\begin{array}{c} Im\left({\bar{V}}_{i,\rho -1}\right)=Im\left(\left[\begin{array}{cc} W_{i,\rho } & {\bar{V}}_{i,\rho } \end{array}\right]\right), \end{array}$$

with ${\bar{V}}_{i,-1}:=I_{n}$.

It is worth pointing out that $Im(W_{i,\rho })$ corresponds to the innovation introduced by the ρ-hop reachable set $N_{i,\rho }$ of agent *i*, that is, the observable modes for agent *i* at hop ρ that are not observable at hop ρ−1. Accordingly, the transformation matrix $T_{i}$, defined as $T_{i}=\left[{\bar{V}}_{i,\ell_{i}}\;\;V_{i,\ell_{i}}\right]$, can be divided using the innovations at each hop:

(7) $$T_{i}:=[\underset{{\bar{V}}_{i,\rho }}{\underset{︸}{\begin{array}{cccc} {\bar{V}}_{i,\ell_{i}} & W_{i,\ell_{i}} & \cdots & W_{i,\rho +1} \end{array}}}\underset{V_{i,\rho }}{\underset{︸}{\begin{array}{ccc} W_{i,\rho } & \cdots & W_{i,0} \end{array}}}]\in R^{n\times n},$$

for all $\rho \in \{0,\cdots ,\ell_{i}\}$, where it is easy to identify the observable and unobservable subspaces of the system by agent *i* at hop ρ.

The following lemma, previously presented in [21], introduces some important properties that are central for the subsequent derivations.

**Lemma** **1.** *For any agent i∈V, the next properties hold, ∀ρ, $\rho^{\prime }\in \left\{1,\cdots ,\ell_{i}\right\}$ such that $\rho \neq \rho^{\prime }$:*

*(i)*  *$W_{i,\rho }^{⊤}W_{i,\rho^{\prime }}=0$,*
*(ii)*  *$Im\left(W_{j,\rho -1}\right)\subseteq Im\left(V_{i,\rho }\right),\;\;\forall j\in N_{i}$,*
*(iii)*  *$Im\left(W_{i,\rho }\right)\subseteq \underset{j\in N_{i}}{⨁}Im\left(W_{j,\rho -1}\right)$,*
*(iv)*  *$Im\left(A{\bar{V}}_{i,\rho }\right)\subseteq Im\left({\bar{V}}_{i,\rho }\right)$.*

**Figure 1:**
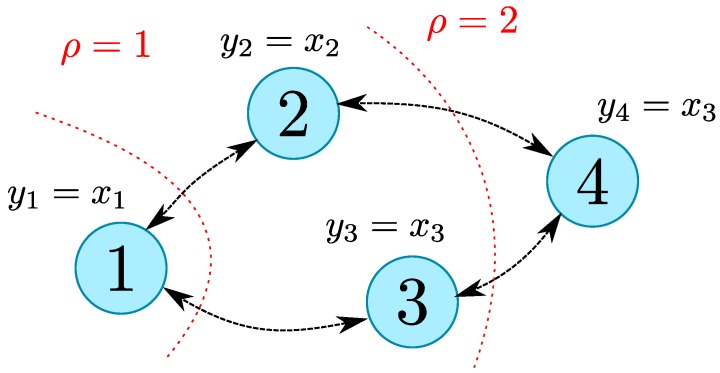
Assume that agent 1 does not want to consider $y_{3}$. Then, it can design $W_{1,1}$ to only include the basis vector of $y_{2}$ and $W_{1,2}$ to include the corresponding basis of $y_{3}$.

**Remark** **1.** *The subspace decomposition can be altered willfully in the case that one agent does not want to consider some measurements (see for instance the example in Figure 1).*

Once the above lemma has been introduced, the following proposition can be established. This proposition is key for the observer to be presented in the next section.

**Proposition** **1.** *For each agent i, the orthogonal similarity transformation given by $T_{i}$ in* (7) *transforms the system matrix A into a block upper-triangular matrix in the form [21]:*

(8) $$T_{i}^{⊤}AT_{i}=\left[\begin{array}{ccccc} {\bar{V}}_{i,\ell_{i}}^{⊤}A{\bar{V}}_{i,\ell_{i}} & {\bar{V}}_{i,\ell_{i}}^{⊤}AW_{i,\ell_{i}} & \cdots & {\bar{V}}_{i,\ell_{i}}^{⊤}AW_{i,1} & {\bar{V}}_{i,\ell_{i}}^{⊤}AW_{i,0}\\ 0 & W_{i,\ell_{i}}^{⊤}AW_{i,\ell_{i}} & \cdots & W_{i,\ell_{i}}^{⊤}AW_{i,1} & W_{i,\ell_{i}}^{⊤}AW_{i,0}\\ \vdots & \vdots & \ddots & \vdots & \vdots \\ 0 & 0 & \cdots & W_{i,1}^{⊤}AW_{i,1} & W_{i,1}^{⊤}AW_{i,0}\\ 0 & 0 & \cdots & 0 & W_{i,0}^{⊤}AW_{i,0} \end{array}\right]$$

The following lemma introduces an important property to be considered.

**Lemma** **2.** *For any i∈V and any $j\in N_{i,\rho }$, it holds $C_{j}W_{i,r}=0$, with $\rho ,r\in \{0,\cdots ,\ell_{i}\}$ and r>ρ.*

**Proof.**  From Definition 1 it is easy to see that $Im\left(C_{j}^{⊤}\right)\subseteq Im\left(C_{i,\rho }^{⊤}\right)$ for all $j\in N_{i,\rho }$. By using Definition 3, we have that pair $\left(C_{i,\rho },A\right)$ generates the subspace $O_{i,\rho }$ which directly implies that $Im(C_{i,\rho }^{⊤})$ belongs to $Im(V_{i,\rho })$ and consequently $Im\left(C_{j}^{⊤}\right)\subseteq Im\left(V_{i,\rho }\right)$. Finally, considering the orthogonality between $V_{i,\rho }$ and $W_{i,r}$ for every r>ρ the Lemma is proved. □

## 3. Observer Structure and Design Goal

This section presents the observer structure that makes use of the notions previously introduced:

(9) $${\hat{x}}_{i}\left(k+1\right)=\underset{\left(a\right)}{\underset{︸}{A{\hat{x}}_{i}\left(k\right)}}+\underset{\left(b\right)}{\underset{︸}{\sum_{\rho =0}^{\ell_{i}}W_{i,\rho }N_{i,\rho }\left(k\right)W_{i,\rho }^{⊤}\sum_{j\in N_{i,\rho }}C_{j}^{⊤}\left(y_{j}\left(k-\rho \right)-C_{j}{\hat{x}}_{i}\left(k-\rho \right)\right)}},$$

where ρ is the distance from agent *i* to the agent that constitutes the source of information, and $N_{i,\rho }\in R^{n_{i,\rho }\times n_{i,\rho }}$ are a set of gains to be designed. Recall that, since we are assuming slow communication networks in which the information consumes one sample time to get from one agent to its neighbors, this information reaches the destination agent with a fixed delay of ρ sample times (The fixed delay for the communication from agent *j* to agent *i* depends on their relative position in the graph. Therefore we could have written $\rho_{i,j}$ instead of ρ. For the sake of simplicity on the notation, we have kept the second option.). In other words, there exists a match between the total delay the packet suffers between sender and receiver, and the hop at which the information affects (It is trivially easy to modify the delay for the communications to other values higher than one. However, this would make the notation harder.).

The observer structure proposed in (9) decomposes the observer dynamics in two different terms:

(a) The first term is the classical model-based open-loop prediction term.
(b) The second one is a correction term. The agents belonging to the ρ−hop reachable set of *i*, $N_{i,\rho }$, communicate the measurements made to agent *i*. Since transmitted measurements flow through the graph at a rate of 1 hop per sampling time, any agent *i* receives the measurements from its ρ-hop reachable set with a constant delay of ρ sampling times. The correction made with these measurements is projected into $Im(W_{i,\rho })$ and multiplied by the gain matrix $N_{i,\rho }\left(k\right)$. The result is used as weights to perform linear combinations of $W_{i,\rho }$. Thus, these corrections only affect the observable subspace generated by the innovations of the neighborhood of agent *i* at hop ρ.

**Remark** **2.** *It is worth pointing out that if $\ell_{i}=0$, pair $\left(C_{i},A\right)$ is observable and consequently $W_{i,0}$ is a full rank matrix. Thus, Equation* (9) *can be rewritten as:*

$${\hat{x}}_{i}\left(k+1\right)=A{\hat{x}}_{i}\left(k\right)+L_{i}\left(k\right)\left(y_{i}\left(k\right)-C_{i}{\hat{x}}_{i}\left(k\right)\right),$$

*where $L_{i}\left(k\right)=W_{i,0}N_{i,0}\left(k\right)W_{i,0}^{⊤}C_{i}^{⊤}$. Note that above expression is clearly the well-known Luenberger observer structure.*

For each agent i∈V, let us define the estimation error as $e_{i}:=x-{\hat{x}}_{i}$, and similarly, the transformed estimation error as $\varepsilon_{i}:=\xi_{i}-{\hat{\xi }}_{i}=T_{i}^{⊤}e_{i}$, which can be decomposed in the transformed estimation error of agent *i* at each hop ρ:

(10) $$\varepsilon_{i}=\left[\begin{array}{c} \varepsilon_{i,\ell_{i}+1}\\ \varepsilon_{i,\ell_{i}}\\ \vdots \\ \varepsilon_{i,1}\\ \varepsilon_{i,0} \end{array}\right]=\left[\begin{array}{c} {\bar{V}}_{i,\ell_{i}}^{⊤}\\ W_{i,\ell_{i}}^{⊤}\\ \vdots \\ W_{i,1}^{⊤}\\ W_{i,0}^{⊤} \end{array}\right]e_{i},$$

and thus, due to the fact that $T_{i}$ is an orthogonal matrix (and therefore $T_{i}^{⊤}T_{i}=I_{n}$), the expression of the estimation error in $\varepsilon_{i,\rho }$ coordinates yields:

(11) $$e_{i}={\bar{V}}_{i,\ell_{i}}\varepsilon_{i,\ell_{i}+1}+\sum_{r=0}^{\ell_{i}}W_{i,r}\varepsilon_{i,r}.$$

The goal of this paper is to design the gain matrices $N_{i,\rho }\left(k\right)$ in structure (9) for every agent *i* and every hop $\rho \in \{0,\cdots ,\ell_{i}\}$ to solve the following problems:

**Problem** **1.** *(Distributed data fusion) In the absence of plant and measurement noises, i.e., w(k)=0 and $n_{i}\left(k\right)=0$, given plant *(1)* and *(2)*, and the interconnection graph G=(V,E), design gains $N_{i,\rho }\left(k\right)$ in *(9)* such that all the estimates ${\hat{x}}_{i}$ asymptotically converge to the actual plant state x.*

**Problem** **2.** *(Distributed optimal filtering) Given plant *(1)* and *(2)* and the interconnection graph G=(V,E), design gains $N_{i,\rho }\left(k\right)$ in *(9)* in order to minimize $E\{||\varepsilon_{i,\rho }\left(k+1\right)|{|}^{2}\}$.*

The solutions for Problems 1 and 2 are introduced in Section 5 and Section 6, respectively. However, prior to that, it is necessary to introduce some properties on which subsequent developments are supported. Additionally, the setup procedure of the distributed observer is presented next.

### Distributed Observer Setup

It is worth mentioning that observer structure (9) needs some neighboring information before starting the estimation phase. That is, the construction of matrices $W_{i,\rho }$ for every $\rho \in \{0,\cdots ,\ell_{i}\}$ and the value of $\ell_{i}$ for every agent i∈V it is needed before starting the estimation procedure.

This section presents an algorithm to design the matrices and parameters aforementioned. Note that although gain matrices $N_{i,\rho }\left(k\right)$ are also required before the estimation phase, this design is tackled in the following sections. The pseudocode of the setup algorithm is given in Algorithm 1.

 **Algorithm 1:** Distributed Observer Setup Algorithm
**1** For every agent *i* do:**2**  Set ρ=0.**3**  Perform the two steps:**4**   Exchange matrices $C_{i,\rho }$ with the neighborhood $N_{i}$.**5**   Compute $C_{i,\rho +1}$ and matrix $W_{i,\rho }$. Include the path in the routing tables of the interconnection nodes.**6**  If pair $\left(C_{i,\rho +1},A\right)$ is detectable, then stop and fix $\ell_{i}=\rho +1$. Otherwise increment ρ and go to 3.


The routing tables of the interconnection nodes includes the information that the agents must know in order to route the information from one source agent to the destination agent in a direct path.

After this initialization phase, that finishes after a finite number of steps, the agents are ready to execute their estimation of the state in a distributed way.

**Remark** **3.** *By letting this algorithm be executed successive times, and not just at the initialization phase, the agents can detect changes in the topology and, then, redesign their observer gains. Hence, the proposed observer can be made resilient to time-varying topologies.*

**Remark** **4.** *Consider a situation in which the destination agent i is selective with the agent who will act as a source of information to reconstruct some part of the state, as was introduced in the Remark 1. In this situation, agent i must take this fact under consideration in step **5** of the setup algorithm, modifying matrices $C_{i,\rho }$ and consequently matrices $W_{i,\rho }$ (which denote the innovation basis of agent i of the observable subspace at hop ρ).*

## 4. Estimation Error Dynamics

This section presents the evolution of the transformed estimation error, $\varepsilon_{i,\rho }\left(k\right)$. After introducing these dynamics, it will become clear that new delayed versions of this error will need to be defined, together with their associate dynamics.

**Proposition** **2.** *Consider the network of agents described by the graph G=(V,E), where every agent i implements the observer structure *(9)* to estimate the state of the system *(1)*. Then, the transformed estimation error dynamics at every hop ρ is given by the following equation:*

(12) $$\begin{array}{ccc} \varepsilon_{i,\rho }\left(k+1\right) & = & W_{i,\rho }^{⊤}A\sum_{r=0}^{\rho }W_{i,r}\varepsilon_{i,r}\left(k\right)+W_{i,\rho }^{⊤}w\left(k\right)\\ & - & N_{i,\rho }\left(k\right)W_{i,\rho }^{⊤}\sum_{j\in N_{i,\rho }}C_{j}^{⊤}\left(C_{j}\sum_{r=0}^{\rho }W_{i,r}\varepsilon_{i,r}\left(k-\rho \right)+n_{j}\left(k-\rho \right)\right), \end{array}$$

*for all $\rho =\{0,\cdots ,\ell_{i}\}$.*

**Proof.**  Let us write first the evolution of the estimation error dynamics for system (1) under the observation structure in (9):

$$\begin{array}{ccc} e_{i}\left(k+1\right) & = & x\left(k+1\right)-{\hat{x}}_{i}\left(k+1\right)\\ & = & Ae_{i}\left(k\right)-\sum_{\rho =0}^{\ell_{i}}W_{i,\rho }N_{i,\rho }\left(k\right)W_{i,\rho }^{⊤}\sum_{j\in N_{i,\rho }}C_{j}^{⊤}\left(C_{j}e_{i}\left(k-\rho \right)+n_{j}\left(k-\rho \right)\right)+w\left(k\right). \end{array}$$

Using (10), we can write the transformed estimation error dynamics for agent *i* at hop ρ:

$$\begin{array}{ccc} \varepsilon_{i,\rho }\left(k+1\right) & = & W_{i,\rho }^{⊤}e_{i}\left(k+1\right)\\ & = & W_{i,\rho }^{⊤}Ae_{i}\left(k\right)-N_{i,\rho }\left(k\right)W_{i,\rho }^{⊤}\sum_{j\in N_{i,\rho }}C_{j}^{⊤}\left(C_{j}e_{i}\left(k-\rho \right)+n_{j}\left(k-\rho \right)\right)+W_{i,\rho }^{⊤}w\left(k\right), \end{array}$$

where $W_{i,\rho }^{⊤}W_{i,\rho }=I_{n_{i,\rho }}$ and Lemma 1 (i) has been used. Next, relying on Equation (11), we know the expression of $e_{i}$ in $\varepsilon_{i,\rho }$ coordinates, so previous equation can be accordingly modified:

$$\begin{array}{c} \varepsilon_{i,\rho }\left(k+1\right)=W_{i,\rho }^{⊤}A\left({\bar{V}}_{i,\ell_{i}}\varepsilon_{i,\ell_{i}+1}\left(k\right)+\sum_{r=0}^{\ell_{i}}W_{i,r}\varepsilon_{i,r}\left(k\right)\right)+W_{i,\rho }^{⊤}w\left(k\right)\\ -N_{i,\rho }\left(k\right)W_{i,\rho }^{⊤}\sum_{j\in N_{i,\rho }}C_{j}^{⊤}\left(C_{j}\left({\bar{V}}_{i,\ell_{i}}\varepsilon_{i,\ell_{i}+1}\left(k-\rho \right)+\sum_{r=0}^{\ell_{i}}W_{i,r}\varepsilon_{i,r}\left(k-\rho \right)\right)+n_{j}\left(k-\rho \right)\right). \end{array}$$

From Lemma 1 (iv) it holds $W_{i,\rho }^{⊤}A\left({\bar{V}}_{i,\ell_{i}}\varepsilon_{i,\ell_{i}+1}+\sum_{r=\rho +1}^{\ell_{i}}W_{i,r}\varepsilon_{i,r}\right)=0$ so we can rewrite the above equation as:

$$\begin{array}{c} \varepsilon_{i,\rho }\left(k+1\right)=W_{i,\rho }^{⊤}A\sum_{r=0}^{\rho }W_{i,r}\varepsilon_{i,r}\left(k\right)+W_{i,\rho }^{⊤}w\left(k\right)\\ -N_{i,\rho }\left(k\right)W_{i,\rho }^{⊤}\sum_{j\in N_{i,\rho }}C_{j}^{⊤}\left(C_{j}{\bar{V}}_{i,\ell_{i}}\varepsilon_{i,\ell_{i}+1}\left(k-\rho \right)+C_{j}\sum_{r=0}^{\ell_{i}}W_{i,r}\varepsilon_{i,r}\left(k-\rho \right)+n_{j}\left(k-\rho \right)\right). \end{array}$$

Next, from Lemma 2 we know that $C_{j}W_{i,\rho^{\prime }}=0$ for all $j\in N_{i,\rho }$ with $\rho^{\prime }>\rho$ and therefore

$$\begin{array}{ccc} \varepsilon_{i,\rho }\left(k+1\right) & = & W_{i,\rho }^{⊤}A\sum_{r=0}^{\rho }W_{i,r}\varepsilon_{i,r}\left(k\right)+W_{i,\rho }^{⊤}w\left(k\right)\\ & - & N_{i,\rho }\left(k\right)W_{i,\rho }^{⊤}\sum_{j\in N_{i,\rho }}C_{j}^{⊤}\left(C_{j}\sum_{r=0}^{\rho }W_{i,r}\varepsilon_{i,r}\left(k-\rho \right)+n_{j}\left(k-\rho \right)\right), \end{array}$$

which is the desired expression. □

Now, let us define the delayed transformed estimation error of agent *i* at hop ρ, ${\bar{\varepsilon }}_{i,\rho }$, as the vector that stacks the transformed estimation error of agent *i* at hop ρ from time *k* to $k-\ell_{i}$, that is:

(13) $${\bar{\varepsilon }}_{i,\rho }\left(k\right):=\left[\begin{array}{c} \varepsilon_{i,\rho }\left(k\right)\\ \varepsilon_{i,\rho }\left(k-1\right)\\ \vdots \\ \varepsilon_{i,\rho }\left(k-\ell_{i}\right) \end{array}\right],$$

and let us define ${\bar{\varepsilon }}_{i}$ as the vector that stacks the delayed transformation error of agent *i* at every hop $\rho \in \{0,\cdots ,\ell_{i}\}$ sorted in decreasing order

(14) $${\bar{\varepsilon }}_{i}\left(k\right):=\left[\begin{array}{c} {\bar{\varepsilon }}_{i,\ell_{i}}\left(k\right)\\ {\bar{\varepsilon }}_{i,\ell_{i}-1}\left(k\right)\\ \vdots \\ {\bar{\varepsilon }}_{i,0}\left(k\right) \end{array}\right].$$

Observe that $\varepsilon_{i,\rho }\left(k\right)=S_{i,\rho }{\bar{\varepsilon }}_{i,\rho }\left(k\right)$, with $S_{i,\rho }=\left[\begin{array}{cc} I_{n_{i,\rho }} & 0\;\;\;\;\cdots \;\;\;\;0 \end{array}\right]\in R^{n_{i,\rho }\times \left(\ell_{i}+1\right)n_{i,\rho }}$. Analogously, $\varepsilon_{i,\rho }\left(k\right)={\bar{S}}_{i,\rho }{\bar{\varepsilon }}_{i}\left(k\right)$, with ${\bar{S}}_{i,\rho }=S_{i,\rho }\left[\begin{array}{ccc} 0\;\;\cdots \;\;0 & I_{\left(\ell_{i}+1\right)n_{i,\rho }} & 0\;\;\;\;\cdots \;\;\;\;0 \end{array}\right]\in R^{n_{i,\rho }\times \sum_{r=0}^{\ell_{i}}\left(\ell_{i}+1\right)n_{i,r}}$.

Based on these error vectors, we can obtain an expression for the dynamics of the delayed transformed estimation error, ${\bar{\varepsilon }}_{i,\rho }$, that will be useful later.

**Proposition** **3.** *Consider the network of agents described by the graph G=(V,E), where every agent i implements the observer structure *(9)* to estimate the state of the system *(1)*. Then, the delayed transformed estimation error dynamics at hop ρ is given by the following equation:*

(15) $${\bar{\varepsilon }}_{i,\rho }\left(k+1\right)=\sum_{r=0}^{\rho }{▵}_{i,(\rho ,r)}\left(k\right){\bar{\varepsilon }}_{i,r}\left(k\right)+S_{i,\rho }^{⊤}W_{i,\rho }^{⊤}w\left(k\right)-S_{i,\rho }^{⊤}N_{i,\rho }\left(k\right)W_{i,\rho }^{⊤}\sum_{j\in N_{i,\rho }}C_{j}^{⊤}n_{j}\left(k-\rho \right),$$

*being*

(16) $${▵}_{i,(\rho ,\rho )}\left(k\right)=\left[\begin{array}{ccccccc} W_{i,\rho }^{⊤}AW_{i,\rho } & 0 & \cdots & -N_{i,\rho }\left(k\right)W_{i,\rho }^{⊤}\sum_{j\in N_{i,\rho }}C_{j}^{⊤}C_{j}W_{i,\rho } & \cdots & 0 & 0\\ I_{n_{i,\rho }} & 0 & \cdots & 0 & \cdots & 0 & 0\\ 0 & I_{n_{i,\rho }} & \cdots & 0 & \cdots & 0 & 0\\ \vdots & \vdots & & \ddots & & \vdots & \vdots \\ 0 & 0 & \cdots & 0 & \cdots & I_{n_{i,\rho }} & 0 \end{array}\right]\in R^{\left(\ell_{i}+1\right)\left(n_{i,\rho }\times n_{i,\rho }\right)},$$

*and*

(17) $${▵}_{i,(\rho ,r)}\left(k\right)=\left[\begin{array}{ccccccc} W_{i,\rho }^{⊤}AW_{i,r} & 0 & \cdots & -N_{i,\rho }\left(k\right)W_{i,\rho }^{⊤}\sum_{j\in N_{i,\rho }}C_{j}^{⊤}C_{j}W_{i,r} & \cdots & 0 & 0\\ 0 & 0 & \cdots & 0 & \cdots & 0 & 0\\ 0 & 0 & \cdots & 0 & \cdots & 0 & 0\\ \vdots & \vdots & & \ddots & & \vdots & \vdots \\ 0 & 0 & \cdots & 0 & \cdots & 0 & 0 \end{array}\right]\in R^{\left(\ell_{i}+1\right)\left(n_{i,\rho }\times n_{i,r}\right)},$$

*for r≠ρ and $r,\rho \in \{0,\cdots ,\ell_{i}\}$ where the terms $-N_{i,\rho }\left(k\right)W_{i,\rho }^{⊤}\sum_{j\in N_{i,\rho }}C_{j}^{⊤}C_{j}W_{i,\rho }$ and $-N_{i,\rho }\left(k\right)W_{i,\rho }^{⊤}\sum_{j\in N_{i,\rho }}C_{j}^{⊤}C_{j}W_{i,r}$ are placed in the block component (1,ρ+1) of the matrices ${▵}_{i,(\rho ,\rho )}$ and ${▵}_{i,(\rho ,r)}$, respectively.*

The proof is immediate by substituting Equation (12) into Equation (13).

## 5. Distributed Data Fusion

This section tackles Problem 1. In particular, the section presents necessary and sufficient stability conditions for the observer structure (9) in absence of perturbations. Moreover, a linear matrix inequality (LMI) method (see [24] for details) for the design of gain matrices $N_{i,\rho }\left(k\right)$ that guarantees stability is introduced. This design is independent of time and then, for simplicity in the notation we will use $N_{i,\rho }=N_{i,\rho }\left(k\right)$.

**Theorem** **1.** *Consider the network of agents described by the graph G=(V,E), where every agent i implements the observer structure *(9)* to estimate the state of the system *(1)*. Under the unperturbed scenario, the estimates of all the agents tend asymptotically to the actual plant state if and only if matrices ${▵}_{i,(\rho ,\rho )}$ are Schur for every $\rho \in \;\{0,\cdots ,\ell_{i}\}$.*

**Proof.**  The dynamics of the delayed transformed estimation error of agent *i* at hop ρ is given in Proposition 3. By setting $w\left(k\right)\equiv n_{i}\left(k\right)=0$, ∀i∈V, in (15), the following expression can be obtained:

(18) $$\underset{{\bar{\varepsilon }}_{i}\left(k+1\right)}{\underset{︸}{\left[\begin{array}{c} {\bar{\varepsilon }}_{i,\ell_{i}}\left(k+1\right)\\ {\bar{\varepsilon }}_{i,\ell_{i}-1}\left(k+1\right)\\ \vdots \\ {\bar{\varepsilon }}_{i,0}\left(k+1\right) \end{array}\right]}}=\left[\begin{array}{cccc} {▵}_{i,(\ell_{i},\ell_{i})} & {▵}_{i,(\ell_{i},\ell_{i}-1)} & \cdots & {▵}_{i,(\ell_{i},0)}\\ 0 & {▵}_{i,(\ell_{i}-1,\ell_{i}-1)} & \cdots & {▵}_{i,(\ell_{i}-1,0)}\\ \vdots & \vdots & \ddots & \vdots \\ 0 & 0 & \cdots & {▵}_{i,(0,0)} \end{array}\right]\underset{{\bar{\varepsilon }}_{i}\left(k\right)}{\underset{︸}{\left[\begin{array}{c} {\bar{\varepsilon }}_{i,\ell_{i}}\left(k\right)\\ {\bar{\varepsilon }}_{i,\ell_{i}-1}\left(k\right)\\ \vdots \\ {\bar{\varepsilon }}_{i,0}\left(k\right) \end{array}\right]}}.$$

Please note that (18) reveals a cascade structure in which the delayed transformed estimation error at each hop ρ depends on the errors at previous hops. Thus, the eigenvalues of the block upper triangular matrix in (18) are given by the eigenvalues of the corresponding matrices placed in its diagonal, which are the matrices defined in (16) establishing the proof. □

It is worth pointing out that the stability of the distributed observer can be seen as the stability of several constant time-delay discrete-time systems. Next, a design method for gain matrices $N_{i,\rho }$ that met Theorem 1 is presented.

**Theorem** **2.** *(Design for stability) If there exist matrices $R_{i,\rho }\in R^{n_{i,\rho }\times n_{i,\rho }}$, $X_{i,\rho }\in R^{n_{i,\rho }\times n_{i,\rho }}$ and $Y_{i,\rho }=\left[\begin{array}{ccc} Y_{i,\rho }^{\left(1,1\right)} & \cdots & Y_{i,\rho }^{\left(1,\ell_{i}\right)}\\ \vdots & \ddots & \vdots \\ Y_{i,\rho }^{\left(\ell_{i},1\right)} & \cdots & Y_{i,\rho }^{\left(\ell_{i},\ell_{i}\right)} \end{array}\right]\in R^{\ell_{i}\left(n_{i,\rho }\times n_{i,\rho }\right)}$ such that the LMI*

(19) $$\begin{array}{c} \left[\begin{array}{cccc} X_{i,\rho }-Y_{i,\rho }^{\left(1,1\right)} & -Y_{i,\rho }^{\left(1,2\right)} & \cdots & -Y_{i,\rho }^{\left(1,\rho +1\right)}\\ -Y_{i,\rho }^{\left(2,1\right)} & Y_{i,\rho }^{\left(1,1\right)}-Y_{i,\rho }^{\left(2,2\right)} & \cdots & Y_{i,\rho }^{\left(1,\rho \right)}-Y_{i,\rho }^{\left(2,\rho +1\right)}\\ \vdots & \vdots & & \vdots \\ -Y_{i,\rho }^{\left(\rho +1,1\right)} & Y_{i,\rho }^{\left(\rho ,1\right)}-Y_{i,\rho }^{\left(\rho +1,2\right)} & \cdots & Y_{i,\rho }^{\left(\rho ,\rho \right)}-Y_{i,\rho }^{\left(\rho +1,\rho +1\right)}\\ \vdots & \vdots & & \vdots \\ -Y_{i,\rho }^{\left(\ell_{i},1\right)} & Y_{i,\rho }^{\left(\ell_{i}-1,1\right)}-Y_{i,\rho }^{\left(\ell_{i},2\right)} & \cdots & Y_{i,\rho }^{\left(\ell_{i}-1,\rho \right)}-Y_{i,\rho }^{\left(\ell_{i},\rho +1\right)}\\ 0 & Y_{i,\rho }^{\left(\ell_{i},1\right)} & \cdots & Y_{i,\rho }^{\left(\ell_{i},\rho \right)}\\ X_{i,\rho }W_{i,\rho }^{⊤}AW_{i,\rho } & 0 & \cdots & -R_{i,\rho }W_{i,\rho }^{⊤}\sum_{j\in N_{i,\rho }}C_{j}^{⊤}C_{j}W_{i,\rho } \end{array}\right.\\ \left.\begin{array}{cccc} \cdots & -Y_{i,\rho }^{\left(1,\ell_{i}\right)} & 0 & W_{i,\rho }^{⊤}A^{⊤}W_{i,\rho }X_{i,\rho }\\ \cdots & Y_{i,\rho }^{\left(1,\ell_{i}-1\right)}-Y_{i,\rho }^{\left(2,\ell_{i}\right)} & Y_{i,\rho }^{\left(1,\ell_{i}\right)} & 0\\ & \vdots & \vdots & \vdots \\ \cdots & Y_{i,\rho }^{\left(\rho ,\ell_{i}-1\right)}-Y_{i,\rho }^{\left(\rho +1,\ell_{i}\right)} & Y_{i,\rho }^{\left(\rho ,\ell_{i}\right)} & -W_{i,\rho }^{⊤}\sum_{j\in N_{i,\rho }}C_{j}^{⊤}C_{j}W_{i,\rho }R_{i,\rho }^{⊤}\\ & \vdots & \vdots & \vdots \\ \cdots & Y_{i,\rho }^{\left(\ell_{i},\ell_{i}-1\right)}-Y_{i,\rho }^{\left(\ell_{i},\ell_{i}\right)} & Y_{i,\rho }^{\left(\ell_{i}-1,\ell_{i}\right)} & 0\\ \cdots & Y_{i,\rho }^{\left(\ell_{i},\ell_{i}-1\right)} & Y_{i,\rho }^{\left(\ell_{i},\ell_{i}\right)} & 0\\ \cdots & 0 & 0 & X_{i,\rho } \end{array}\right]>0, \end{array}$$

*is satisfied, then gain $N_{i,\rho }=R_{i,\rho }X_{i,\rho }^{-1}$ stabilizes matrices *(16)*, and therefore the estimates of every agent tends asymptotically to the actual plant state.*

**Proof.**  From Lyapunov theory [24], Theorem 1 is fulfilled if and only if there exists a positive definite matrix $P_{i,\rho }\in R^{\left(\ell_{i}+1\right)\left(n_{i,\rho }\times n_{i,\rho }\right)}$ such that:

$$\begin{array}{c} P_{i,\rho }>0,\\ P_{i,\rho }-{▵}_{i,(\rho ,\rho )}^{⊤}P_{i,\rho }{▵}_{i,(\rho ,\rho )}>0. \end{array}$$

Now, define $P_{i,\rho }=\;\left[\begin{array}{cc} X_{i,\rho } & 0\\ 0 & Y_{i,\rho } \end{array}\right]$ with $X_{i,\rho }\in R^{n_{i,\rho }\times n_{i,\rho }}$ and $Y_{i,\rho }\in R^{\ell_{i}\left(n_{i,\rho }\times n_{i,\rho }\right)}$.

With some mathematical manipulations, previous conditions can be rewritten as

$$\begin{array}{c} 0<\left[\begin{array}{cccc} X_{i,\rho } & 0 & \cdots & 0\\ 0 & Y_{i,\rho }^{\left(1,1\right)} & \cdots & {\mathrm{error}}_{i,\rho }^{\left(1,\ell_{i}\right)}\\ \vdots & \vdots & \ddots & \vdots \\ 0 & Y_{i,\rho }^{\left(\ell_{i},1\right)} & \cdots & Y_{i,\rho }^{\left(\ell_{i},\ell_{i}\right)} \end{array}\right]-\left[\begin{array}{cccc} Y_{i,\rho }^{\left(1,1\right)} & \cdots & Y_{i,\rho }^{\left(1,\ell_{i}\right)} & 0\\ \vdots & \ddots & \vdots & 0\\ Y_{i,\rho }^{\left(\ell_{i},1\right)} & \cdots & Y_{i,\rho }^{\left(\ell_{i},\ell_{i}\right)} & 0\\ 0 & \cdots & 0 & 0 \end{array}\right]\\ -\left[\begin{array}{c} W_{i,\rho }^{⊤}A^{⊤}W_{i,\rho }\\ 0\\ \vdots \\ -W_{i,\rho }^{⊤}\sum_{j\in N_{i,\rho }}C_{j}^{⊤}C_{j}W_{i,\rho }N_{i,\rho }^{⊤}\\ \vdots \\ 0 \end{array}\right]X_{i,\rho }\left[\begin{array}{cccccc} W_{i,\rho }^{⊤}AW_{i,\rho } & 0 & \cdots & -N_{i,\rho }W_{i,\rho }^{⊤}\sum_{j\in N_{i,\rho }}C_{j}^{⊤}C_{j}W_{i,\rho } & \cdots & 0 \end{array}\right]. \end{array}$$

Adding the first two matrices and applying the Schur complement [24], previous inequality is equivalent to

$$\begin{array}{c} \left[\begin{array}{cccc} X_{i,\rho }-Y_{i,\rho }^{\left(1,1\right)} & -Y_{i,\rho }^{\left(1,2\right)} & \cdots & -Y_{i,\rho }^{\left(1,\rho +1\right)}\\ -Y_{i,\rho }^{\left(2,1\right)} & Y_{i,\rho }^{\left(1,1\right)}-Y_{i,\rho }^{\left(2,2\right)} & \cdots & Y_{i,\rho }^{\left(1,\rho \right)}-Y_{i,\rho }^{\left(2,\rho +1\right)}\\ \vdots & \vdots & & \vdots \\ -Y_{i,\rho }^{\left(\rho +1,1\right)} & Y_{i,\rho }^{\left(\rho ,1\right)}-Y_{i,\rho }^{\left(\rho +1,2\right)} & \cdots & Y_{i,\rho }^{\left(\rho ,\rho \right)}-Y_{i,\rho }^{\left(\rho +1,\rho +1\right)}\\ \vdots & \vdots & & \vdots \\ -Y_{i,\rho }^{\left(\ell_{i},1\right)} & Y_{i,\rho }^{\left(\ell_{i}-1,1\right)}-Y_{i,\rho }^{\left(\ell_{i},2\right)} & \cdots & Y_{i,\rho }^{\left(\ell_{i}-1,\rho \right)}-Y_{i,\rho }^{\left(\ell_{i},\rho +1\right)}\\ 0 & Y_{i,\rho }^{\left(\ell_{i},1\right)} & \cdots & Y_{i,\rho }^{\left(\ell_{i},\rho \right)}\\ W_{i,\rho }^{⊤}AW_{i,\rho } & 0 & \cdots & -N_{i,\rho }W_{i,\rho }^{⊤}\sum_{j\in N_{i,\rho }}C_{j}^{⊤}C_{j}W_{i,\rho } \end{array}\right.\\ \left.\begin{array}{cccc} \cdots & -Y_{i,\rho }^{\left(1,\ell_{i}\right)} & 0 & W_{i,\rho }^{⊤}A^{⊤}W_{i,\rho }\\ \cdots & Y_{i,\rho }^{\left(1,\ell_{i}-1\right)}-Y_{i,\rho }^{\left(2,\ell_{i}\right)} & Y_{i,\rho }^{\left(1,\ell_{i}\right)} & 0\\ & \vdots & \vdots & \vdots \\ \cdots & Y_{i,\rho }^{\left(\rho ,\ell_{i}-1\right)}-Y_{i,\rho }^{\left(\rho +1,\ell_{i}\right)} & Y_{i,\rho }^{\left(\rho ,\ell_{i}\right)} & -W_{i,\rho }^{⊤}\sum_{j\in N_{i,\rho }}C_{j}^{⊤}C_{j}W_{i,\rho }N_{i,\rho }^{⊤}\\ & \vdots & \vdots & \vdots \\ \cdots & Y_{i,\rho }^{\left(\ell_{i},\ell_{i}-1\right)}-Y_{i,\rho }^{\left(\ell_{i},\ell_{i}\right)} & Y_{i,\rho }^{\left(\ell_{i}-1,\ell_{i}\right)} & 0\\ \cdots & Y_{i,\rho }^{\left(\ell_{i},\ell_{i}-1\right)} & Y_{i,\rho }^{\left(\ell_{i},\ell_{i}\right)} & 0\\ \cdots & 0 & 0 & X_{i,\rho }^{-1} \end{array}\right]>0. \end{array}$$

Note that the obtained matrix inequality is not an LMI because there are terms on $X_{i,\rho }$ and $X_{i,\rho }^{-1}$. If the matrix inequality is pre-multiplied and post-multiplied by the symmetric, non-singular matrix $\left[\begin{array}{cc} I_{\left(\ell_{i}+1\right)n_{i,\rho }} & 0\\ 0 & X_{i,\rho } \end{array}\right]$, and by using the change of variables $R_{i,\rho }=N_{i,\rho }X_{i,\rho }$, LMI (19) is obtained, establishing the proof. □

Recall that LMI (19) can be solved locally by every agent, requiring just the information available after the setup described in Section 3.1. The choice of a block-diagonal Lyapunov matrix introduces conservatism, but it is required to transform the matrix inequality into an LMI. Otherwise, the previous condition would be necessary and sufficient for stabilization.

## 6. Distributed Optimal Filtering

This section deals with Problem 2, that is, gains $N_{i,\rho }\left(k\right)$ must be designed in such a way that the expected value of the norm of the delayed transformed estimation error of agent *i* at hop ρ, that is, $E\{||\varepsilon_{i,\rho }\left(k+1\right)|{|}^{2}\}$, is minimized. Note that $E\{||\varepsilon_{i,\rho }{\left(k+1\right)||}^{2}\}=E\left\{\varepsilon_{i,\rho }^{⊤}\left(k+1\right)\varepsilon_{i,\rho }\left(k+1\right)\right\}=\mathrm{tr}\left(E\left\{\varepsilon_{i,\rho }\left(k+1\right)\varepsilon_{i,\rho }^{⊤}\left(k+1\right)\right\}\right)=\mathrm{tr}\left(E\left\{Q_{i,\rho }\left(k+1\right)\right\}\right)$, where $Q_{i,\rho }$ is the covariance matrix of the transformed estimation error of agent *i* at hop ρ. The dynamics of the covariance matrix will be studied and, then, it will be possible to present a method to find the gains $N_{i,\rho }\left(k\right)$ to minimize $\mathrm{tr}(E\left\{Q_{i,\rho }\left(k+1\right)\right\})$.

Let ${\bar{Q}}_{i,(\rho ,r)}\left(k\right)=E\left\{{\bar{\varepsilon }}_{i,\rho }\left(k\right){\bar{\varepsilon }}_{i,r}^{⊤}\left(k\right)\right\}$ be the cross covariance matrix of the delayed transformed estimation error of agent *i* between hops ρ and *r*. Thus, it is possible to conform the covariance matrix of the delayed transformed estimation error of agent *i* for every hop ρ:

$$\begin{array}{ccc} {\bar{Q}}_{i}\left(k\right) & = & E\{{\bar{\varepsilon }}_{i}\left(k\right){\bar{\varepsilon }}_{i}{\left(k\right)}^{⊤}\}\\ & = & E\left\{\left[\begin{array}{cccc} {\bar{\varepsilon }}_{i,\ell_{i}}\left(k\right){\bar{\varepsilon }}_{i,\ell_{i}}{\left(k\right)}^{⊤} & {\bar{\varepsilon }}_{i,\ell_{i}}\left(k\right){\bar{\varepsilon }}_{i,\ell_{i}-1}{\left(k\right)}^{⊤} & \cdots & {\bar{\varepsilon }}_{i,\ell_{i}}\left(k\right){\bar{\varepsilon }}_{i,0}{\left(k\right)}^{⊤}\\ {\bar{\varepsilon }}_{i,\ell_{i}-1}\left(k\right){\bar{\varepsilon }}_{i,\ell_{i}}{\left(k\right)}^{⊤} & {\bar{\varepsilon }}_{i,\ell_{i}-1}\left(k\right){\bar{\varepsilon }}_{i,\ell_{i}-1}{\left(k\right)}^{⊤} & \cdots & {\bar{\varepsilon }}_{i,\ell_{i}-1}\left(k\right){\bar{\varepsilon }}_{i,0}{\left(k\right)}^{⊤}\\ \vdots & \vdots & \ddots & \vdots \\ {\bar{\varepsilon }}_{i,0}\left(k\right){\bar{\varepsilon }}_{i,\ell_{i}}{\left(k\right)}^{⊤} & {\bar{\varepsilon }}_{i,0}\left(k\right){\bar{\varepsilon }}_{i,\ell_{i}-1}{\left(k\right)}^{⊤} & \cdots & {\bar{\varepsilon }}_{i,0}\left(k\right){\bar{\varepsilon }}_{i,0}{\left(k\right)}^{⊤} \end{array}\right]\right\}\\ & = & \left[\begin{array}{cccc} {\bar{Q}}_{i,(\ell_{i},\ell_{i}-1)}\left(k\right) & {\bar{Q}}_{i,(\ell_{i},\ell_{i})}\left(k\right) & \cdots & {\bar{Q}}_{i,(\ell_{i},0)}\left(k\right)\\ {\bar{Q}}_{i,(\ell_{i}-1,\ell_{i}-1)}\left(k\right) & {\bar{Q}}_{i,(\ell_{i}-1,\ell_{i})}\left(k\right) & \cdots & {\bar{Q}}_{i,(\ell_{i}-1,0)}\left(k\right)\\ \vdots & \vdots & \ddots & \vdots \\ {\bar{Q}}_{i,(0,\ell_{i}-1)}\left(k\right) & {\bar{Q}}_{i,(0,\ell_{i})}\left(k\right) & \cdots & {\bar{Q}}_{i,(0,0)}\left(k\right) \end{array}\right]. \end{array}$$

It is worth pointing out that it is possible to relate the diagonal terms of the covariance matrix ${\bar{Q}}_{i}\left(k\right)$ through the use of the selection matrices defined in Section 4. Therefore, $Q_{i,\rho }\left(k\right)=S_{i,\rho }{\bar{Q}}_{i,(\rho ,\rho )}\left(k\right)S_{i,\rho }^{⊤}={\bar{S}}_{i,\rho }{\bar{Q}}_{i}\left(k\right){\bar{S}}_{i,\rho }^{⊤}$.

Note that matrices ${▵}_{i,(\rho ,r)}$ and ${▵}_{i,(\rho ,\rho )}$ in (16) and (17) for $r,\rho \in \{0,\cdots ,\ell_{i}\}$ can be rewritten as:

$${▵}_{i,(\rho ,r)}\left(k\right)={\tilde{▵}}_{i,(\rho ,r)}-S_{i,\rho }^{⊤}N_{i,\rho }\left(k\right)F_{i,(\rho ,r)},$$

where $F_{i,(\rho ,r)}=\left[\begin{array}{cccccc} 0 & 0 & \cdots & W_{i,\rho }^{⊤}\sum_{j\in N_{i,\rho }}C_{j}^{⊤}C_{j}W_{i,r} & \cdots & 0 \end{array}\right]\in R^{n_{i,\rho }\times \left(\ell_{i}+1\right)n_{i,r}}$ and

▵˜i,(ρ,ρ)=Wi,ρ⊤AWi,ρ0⋯00Ini,ρ0⋯00⋮⋱⋮00⋯Ini,ρ0,▵˜i,(ρ,r)=Wi,ρ⊤AWi,r0⋯0000⋯00⋮⋮⋱⋮00⋯00,

for every $r,\rho \in \{0,\cdots ,\ell_{i}\}$ with r≠ρ. Using this decomposition, the next proposition studies the dynamics of the complete covariance matrix ${\bar{Q}}_{i}$. Its proof requires just some simple mathematical manipulations and, hence, it is omitted.

**Proposition** **4.** *The evolution of covariance matrix ${\bar{Q}}_{i}$ is given by:*

(20) $$\begin{array}{ccc} {\bar{Q}}_{i}\left(k+1\right) & = & \left({\tilde{▵}}_{i}-S_{i}^{⊤}N_{i}\left(k\right)F_{i}\right){\bar{Q}}_{i}\left(k\right){\left({\tilde{▵}}_{i}-S_{i}^{⊤}N_{i}\left(k\right)F_{i}\right)}^{⊤}+S_{i}^{⊤}V_{i,\ell_{i}}^{⊤}MV_{i,\ell_{i}}S_{i}\\ & - & S_{i}^{⊤}N_{i}\left(k\right)D_{i}N_{i}^{⊤}\left(k\right)S_{i}, \end{array}$$

*where $N_{i}\left(k\right)=diag{\left(N_{i,\rho }\left(k\right)\right)}_{\rho \in \{\ell_{i},\cdots ,0\}}$, $D_{i}=diag{\left(W_{i,\rho }\sum_{j\in N_{i,\rho }}C_{j}^{⊤}R_{j}C_{j}W_{i,\rho }^{⊤}\right)}_{\rho \in \{\ell_{i},\cdots ,0\}}$, and:*

$$\begin{array}{c} {\tilde{▵}}_{i}=\left[\begin{array}{cccc} {\tilde{▵}}_{i,(\ell_{i},\ell_{i})} & {\tilde{▵}}_{i,(\ell_{i},\ell_{i}-1)} & \cdots & {\tilde{▵}}_{i,(\ell_{i},0)}\\ 0 & {\tilde{▵}}_{i,(\ell_{i}-1,\ell_{i}-1)} & \cdots & {\tilde{▵}}_{i,(\ell_{i}-1,0)}\\ \vdots & \vdots & \ddots & \vdots \\ 0 & 0 & \cdots & {\tilde{▵}}_{i,(0,0)} \end{array}\right],\;\;S_{i}=\left[\begin{array}{c} {\bar{S}}_{i,\ell_{i}}\\ {\bar{S}}_{i,\ell_{i}-1}\\ \vdots \\ {\bar{S}}_{i,0} \end{array}\right],\\ F_{i}=\left[\begin{array}{cccc} F_{i,(\ell_{i},\ell_{i})} & F_{i,(\ell_{i},\ell_{i}-1)} & \cdots & F_{i,(\ell_{i},0)}\\ 0 & F_{i,(\ell_{i}-1,\ell_{i}-1)} & \cdots & F_{i,(\ell_{i}-1,0)}\\ \vdots & \vdots & \ddots & \vdots \\ 0 & 0 & \cdots & F_{i,(0,0)} \end{array}\right], \end{array}$$

Based on this proposition we present a design method for gains $N_{i,\rho }\left(k\right)$ that solves the Problem 2.

**Theorem** **3.** *The set of matrices $N_{i,\rho }\left(k\right)$ that minimize $tr\left(Q_{i,\rho }\left(k+1\right)\right)$ are the ones that solve the following system of equations:*

(21) $$N_{i,\rho }\left(k\right)\left(W_{i,\rho }^{⊤}\sum_{j\in N_{i,\rho }}C_{j}^{⊤}R_{j}C_{j}W_{i,\rho }-F_{i,(\rho ,:)}{\bar{Q}}_{i}\left(k\right)F_{i,(\rho ,:)}^{⊤}\right)={\bar{S}}_{i,\rho }{\tilde{▵}}_{i}{\bar{Q}}_{i}\left(k\right)F_{i,(\rho ,:)}^{⊤},$$

*for every $\rho \in \{0,\cdots ,\ell_{i}\}$, where $F_{i,(\rho ,:)}=\left[0\;\;\cdots \;\;F_{i,(\rho ,\rho )}\;\;\cdots \;\;F_{i,(\rho ,0)}\right]$ denotes the row block $\ell_{i}-\;\rho +1$ of matrix $F_{i}$.*

**Proof.**  The proof is divided into several steps. Firstly, the dynamics of matrix $Q_{i,\rho }$ will be obtained derived from results in Proposition 4. Then, its trace will be derived with respect to $N_{i,\rho }\left(k\right)$ in order to find the value of the gain that minimizes the trace of $Q_{i,\rho }\left(k\right)$.

Observe that $Q_{i,\rho }\left(k\right)={\bar{S}}_{i,\rho }{\bar{Q}}_{i}\left(k\right){\bar{S}}_{i,\rho }^{⊤}$. Then, it holds:

$$\begin{array}{ccc} Q_{i,\rho }\left(k+1\right) & = & \left({\bar{S}}_{i,\rho }{\tilde{▵}}_{i}-{\bar{S}}_{i,\rho }S_{i}^{⊤}N_{i}\left(k\right)F_{i}\right){\bar{Q}}_{i}\left(k\right){\left({\bar{S}}_{i,\rho }{\tilde{▵}}_{i}-{\bar{S}}_{i,\rho }S_{i}^{⊤}N_{i}\left(k\right)F_{i}\right)}^{⊤}\\ & + & {\bar{S}}_{i,\rho }S_{i}^{⊤}V_{i,\ell_{i}}^{⊤}MV_{i,\ell_{i}}S_{i}{\bar{S}}_{i,\rho }^{⊤}-{\bar{S}}_{i,\rho }S_{i}^{⊤}N_{i}\left(k\right)D_{i}N_{i}^{⊤}\left(k\right)S_{i}{\bar{S}}_{i,\rho }^{⊤}. \end{array}$$

It is a simple matter to check that ${\bar{S}}_{i,\rho }S_{i}^{⊤}V_{i,\ell_{i}}^{⊤}=W_{i,\rho }^{⊤}$ and ${\bar{S}}_{i,\rho }S_{i}^{⊤}N_{i}\left(k\right)F_{i}=N_{i,\rho }\left(k\right)F_{i,(\rho ,:)}$, and, consequently, we can rewrite the above expression as:

$$\begin{array}{ccc} Q_{i,\rho }\left(k+1\right) & = & \left({\bar{S}}_{i,\rho }{\tilde{▵}}_{i}-N_{i,\rho }\left(k\right)F_{i,(\rho ,:)}\right){\bar{Q}}_{i}\left(k\right){\left({\bar{S}}_{i,\rho }{\tilde{▵}}_{i}-N_{i,\rho }\left(k\right)F_{i,(\rho ,:)}\right)}^{⊤}\\ & + & W_{i,\rho }^{⊤}MW_{i,\rho }-N_{i,\rho }\left(k\right)W_{i,\rho }^{⊤}\sum_{j\in N_{i,\rho }\left(k\right)}C_{j}^{⊤}R_{j}C_{j}W_{i,\rho }N_{i,\rho }^{⊤}\left(k\right). \end{array}$$

Now, with the purpose of minimizing the trace of the covariance matrix, $tr(Q_{i,\rho }\left(k+1\right))$ is partially derived with respect to the gain matrix $N_{i,\rho }\left(k\right)$:

$$\begin{array}{ccc} \frac{\partial tr(Q_{i,\rho }\left(k+1\right))}{\partial N_{i,\rho }\left(k\right)} & = & -2{\bar{S}}_{i,\rho }{\tilde{▵}}_{i}{\bar{Q}}_{i}\left(k\right)F_{i,(\rho ,:)}^{⊤}+2N_{i,\rho }\left(k\right)F_{i,(\rho ,:)}{\bar{Q}}_{i}\left(k\right)F_{i,(\rho ,:)}^{⊤}\\ & - & 2N_{i,\rho }\left(k\right)W_{i,\rho }^{⊤}\sum_{j\in N_{i,\rho }}C_{j}^{⊤}R_{j}C_{j}W_{i,\rho }. \end{array}$$

Then, equaling the above expression to zero, we can find that the gain $N_{i,\rho }\left(k\right)$ that minimizes the trace of $Q_{i,\rho }\left(k+1\right)$ satisfies

$${\bar{S}}_{i,\rho }{\tilde{▵}}_{i}{\bar{Q}}_{i}\left(k\right)F_{i,(\rho ,:)}^{⊤}+N_{i,\rho }\left(k\right)F_{i,(\rho ,:)}{\bar{Q}}_{i}\left(k\right)F_{i,(\rho ,:)}^{⊤}=N_{i,\rho }\left(k\right)W_{i,\rho }^{⊤}\sum_{j\in N_{i,\rho }}C_{j}^{⊤}R_{j}C_{j}W_{i,\rho }.$$

After some manipulations, expression (21) is found. □

This theorem has proposed a design method for gain matrices $N_{i,\rho }\left(k\right)$ that minimize the expected value of the estimation error. Additionally, the design method only requires solving a system of linear equations that depends only on local information of the agent, allowing its resolution in a distributed way.

The algorithm that every agent i∈V must follow to initialize the distributed estimation procedure and run the estimation phase is summarized in Algorithm 2.

 **Algorithm 2:** Iterative algorithm.

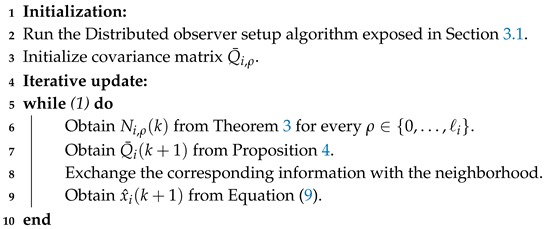



## 7. Simulations Results

In order to show the effectiveness and optimality of the distributed observer some simulations are driven in this section. Consider the following system where there is one state with a stable dynamics, a pair of conjugated imaginary poles and a state with an unstable dynamics:

(22) $$\left[\begin{array}{c} x_{1}\left(k+1\right)\\ x_{2}\left(k+1\right)\\ x_{3}\left(k+1\right)\\ x_{4}\left(k+1\right) \end{array}\right]=\left[\begin{array}{cccc} 0.95 & 0 & 0 & 0\\ 0 & 0.8606 & -1.3368 & 0\\ 0 & 0.0941 & 0.9315 & 0\\ 0 & 0 & 0 & 1.015 \end{array}\right]\left[\begin{array}{c} x_{1}\left(k\right)\\ x_{2}\left(k\right)\\ x_{3}\left(k\right)\\ x_{4}\left(k\right) \end{array}\right].$$

**Figure 2:**
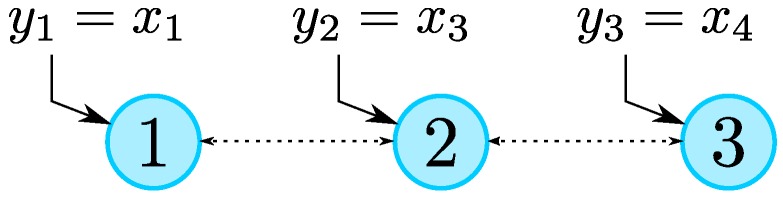
Network topology considered.

The system is being observed by a set of three agents with the network topology depicted in Figure 2.

**Example** **1.** *In this example the performance of the observer is tested under the perturbed scenario in which the system model and the agents measurements are affected by Gaussian noises. Consider the sequel covariance matrices of the noises terms:*

$$M=\left[\begin{array}{cccc} 0.02 & 0 & 0 & 0\\ 0 & 0.05 & 0 & 0\\ 0 & 0 & 0.06 & 0\\ 0 & 0 & 0 & 0.01 \end{array}\right],\;\;\;\;R_{1}=0.01,\;\;\;\;R_{2}=0.02,\;\;\;\;R_{3}=0.01.$$

**Figure 3:**
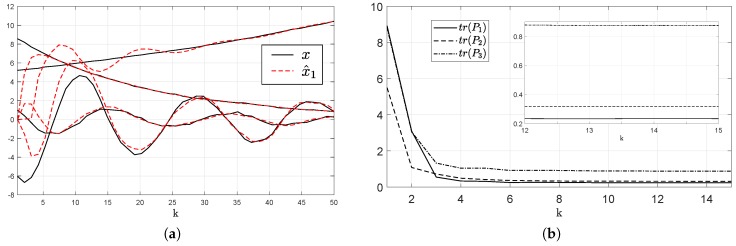
Results of Example 1. (**a**) Evolution of the system state and agent 1 estimates (in dashed lines); (**b**) Evolution of $tr(Q_{i,\rho })$ for every agent.

Figure 3b depicts the evolution of the system state and agent 1 estimates (in dashed lines). It is shown that the estimate of the stable pole that is locally measured by agent 1 converges the first to the real value. After it, the estimates of the states 2 and 3, measured by agent 2, reach the actual value. Finally, the estimator converges to the values of the unstable pole, measured by agent 3 (that belongs to $N_{i,2}$). This simple example makes it clear that the decay rate of the estimator error decreases with ρ. In Figure 3a the evolution of $tr(Q_{i,\rho })$ for every i∈V are shown.

To conclude this section, it is shown that the gain matrices obtained in this example in steady state (through the use of Theorem 3), fulfill the LMI stated in Theorem 2 and, consequently, meet the stability conditions required in Theorem 1. Considering for example agent 1 with the gain matrices $N_{1,\rho }$ obtained through Theorem 3, it is possible to find a solution for the LMI (19) on the variables $X_{1,\rho }$ and $Y_{1,\rho }$, and therefore to guarantee the stability of the estimation errors. The values obtained for $N_{1,\rho }$, $X_{i,\rho }$, and $Y_{1,\rho }$ are given in Table 1.

## 8. Conclusions

In this paper, we present a data-fusion-based algorithm to solve the problem of distributedly estimating the state of a plant through an interconnected network of agents. Based on a coordinate transformation matrix, it is possible to transform the original representation of the state estimation of every agent into the observability staircase form. In this form, the observable modes of each agent at every hop ρ are distinguished. Relying on this transformation, a novel observer structure is presented in such a way that the observer gains can be designed distributedly guaranteeing the stability and minimizing the expected value of the estimation error.

Furthermore, the agents can modify conveniently the construction of the transformation matrix that allows them to perform the subspace decomposition. Thus, they can be selective with those that can act as a source of information of a specific portion of the state.

As a further work it will be interesting to explore the performance of the distributed observer dealing with random events such as packet losses or time-varying delays.

## Figures and Tables

**Table 1:** Values of $X_{1,\rho }$, $Y_{1,\rho }$ and $N_{1,\rho }$ obtained in Theorem 3 for agent 1.

	Agent 1		
	$X_{1,\rho }$	$Y_{1,\rho }$	$N_{1,\rho }$
ρ=0	64.846	$\left[\begin{array}{cc} 57.643 & 0\\ 0 & 41.431 \end{array}\right]$	0.577
ρ=1	$\left[\begin{array}{cc} 0.801 & 0.218\\ 0.218 & 1.090 \end{array}\right]$	$\left[\begin{array}{cccc} 2.165 & 0.435 & 0.671 & -0.033\\ 0.435 & 2.472 & -0.289 & -0.002\\ 0.671 & -0.289 & 2.405 & 0.075\\ -0.033 & -0.002 & 0.075 & 2.909 \end{array}\right]$	$\left[\begin{array}{cc} 0.363 & 0\\ -1.274 & 0 \end{array}\right]$
ρ=2	38.425	$\left[\begin{array}{cc} 44.862 & 0\\ 0 & 51.814 \end{array}\right]$	0.418
